# The complete mitochondrial genome of *Brevimulticaecum sinensis* (Nematoda: Heterocheilidae), the first representative of the genus *Brevimulticaecum*

**DOI:** 10.1080/23802359.2026.2688608

**Published:** 2026-06-19

**Authors:** Jinhong Zhao, Tellez Marisa, Genjun Tu, Armand Kuris, Xiaobing Wu

**Affiliations:** a Department of Medical Parasitology, Wannan Medical College, Wuhu, Anhui, China; b Crocodile Research Coalition, Placencia Peninsula, Stann Creek, Belize; c The National Nature Reserve of China Alligator in Anhui, Xuanzhou, Anhui, China; d Department of Ecology Evolution and Marine Biology and Biology Faculty, College of Creative Studies, University of California, Santa Barbara, California, USA; e Provincial Laboratory of Conservation and Exploitation of Biological Resources, College of Life Sciences, Anhui Normal University, Wuhu, Anhui, China

**Keywords:** *Brevimulticaecum sinensis*, mitochondrial genome, ascaridida nematodes, phylogenetic analysis

## Abstract

The complete mitochondrial genome sequence of *Brevimulticaecum sinensis* was clarified in this study, and this is the first member of the genus *Brevimulticaecum* ever sequenced for complete mitochondrial genome. The circular mitochondrial genome (13887 bp) of *B. sinensis* encodes 12 protein-coding genes, 22 transfer RNA genes, 2 ribosomal RNA genes, and 2 non-coding regions, all genes exhibiting a nucleotide composition bias toward AT. Phylogenetic studies showed that *B. sinensis* and *Ortleppascaris sinensis* formed a monophyletic group belonging to the family Heterocheilidae. The complete mitochondrial genome sequence of *B. sinensis* should enrich molecular markers for studying the molecular classification, genetic variation, and phylogenetic studies of the Ascaridida nematodes.

## Introduction

The genus *Brevimulticaecum,* a member of the superfamily Ascaridoidea, is a major parasitic genus that infects hosts across multiple taxa: Crocodylia including *Alligator*, *Caiman*, *Melanosuchus,* and *Crocodilus*; Osteoglossiformes such as *Heterotis* and *Scleropages*; *and* Myliobatiformes represented by *Potamotrygon.* To date, a total of eleven valid species of *Brevimulticaecum* have been recorded, including *B. baylisi*, *B. tenuicolle*, *B. stekhoveni*, *B. gibsoni*, *B. pintoi*, *B. regoi*, *B. heterotis*, *B. scleropagi*, *B. vandenbrandeni*, *B. australiensis and B. sinensis* (Sprent 1979, Sprent 1990, Junker et al. 2006, Vieira et al. 2010, Zhao et al. 2026). However, no complete mitochondrial genome of genus *Brevimulticaecum* had been documented to date. The reason may be that studies on the nematode *Brevimulticaecum* are scarce due to the difficulty of collecting nematode samples from their hosts and the insufficiency of samples (Sprent 1979, Santana et al. 2023, Zhao et al. 2026). *Brevimulticaecum sinensis* was isolated from the stomach of a carcass of the captive Chinese alligator *Alligator sinensis* (Crocodilian: Alligatoridae) in China by the authors Zhao et al. and named a new species of the genus *Brevimulticaecum* (Zhao et al. 2026). To address this gap, this study sequenced the complete mitochondrial genome of *B. sinensis* and constructed a phylogenetic tree based on 12 protein-coding genes using maximum-likelihood (ML) methods, exploring the phylogenetic relationship between *B. sinensis* and other Ascaridida nematodes. The mitochondrial genome sequence of this nematode can provide useful genetic markers for studying the molecular ecology, phylogenetics and population genetics of the *Brevimulticaecum* nematodes.

## Materials and methods

### Sample collection and DNA extraction

**Figure 1.**
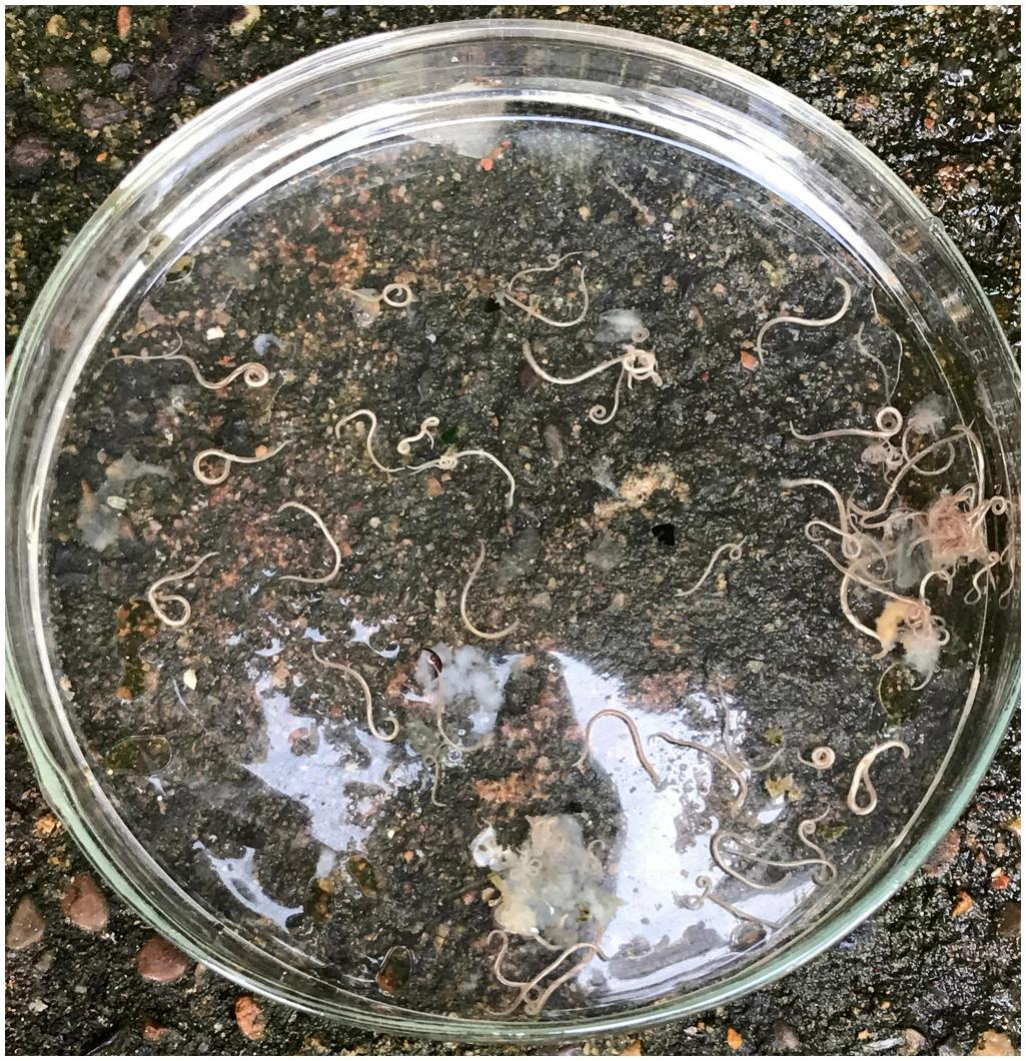
The specimen of *Brevimulticaecum sinensis*, photographed by Jinhong Zhao. Living nematodes were oyster-white in color and moved rapidly forward by twisting their bodies. Males were smaller than females. Based on 10 mature individuals, body length ranged from 13.06 to 15.61 mm for males and from 14.65 to 19.55 mm for females.

Adult nematodes specimen of *B. sinensis* (Figure 1) were obtained from the stomach of the Chinese alligator *A. sinensis* (Crocodilian: Alligatoridae) in the National Nature Reserve of China Alligator (Chinese Crocodile Lake) in Anhui Province, China (30°94′ N, 118°79′ E), In April 2017. Species identification was primarily based on the morphological and molecular characteristics, and named to associate with the genitive form of the host name of *A. sinensis*. (Zhao et al. 2026). The sequenced specimen in this study is from the same population. This study was approved by the Institutional Animal Care and Use Committee of the Wannan Medical College (#LLSC-2024-121). The samples were preserved in 95% ethanol and stored at −20 °C in the department of Parasitology, Wannan Medical College (31°29′ N, 118°37′ E) under the voucher number WNMC-B-110-113 (contact person: Jinhong Zhao, email: zhaojh@wnmc.edu.cn) for future research applications. Total genomic DNA was isolated from individual nematodes using a TIANamp Genomic DNA Kit (TIANGEN, Beijing, China) in accordance with the manufacturer’s protocol. PCR products were electrophoresed in 1% agarose gels, excised, purified using a Column DNA Gel Extraction Kit (Sangon Biotech, Shanghai, China), and subjected to sequencing *via* primer walking with designed species-specific primers (Table S1).

### Sequence analysis and annotation

The nucleotide sequences obtained were assembled manually and aligned, then compared with known sequences in GenBank database using Clustal X 1.83. Protein coding genes (PCGs) were implemented at the NCBI website with the invertebrate mitochondrial genetic code as well as aligning their similarity against the published mitochondrial sequences of Ascaridida nematodes using Clustal X 2.0 and MEGA 7.0. Genes for large subunits ribosomal RNA (*rrn*L) and small subunits ribosomal RNA (*rrn*S) were identified by comparison with the mitochondrial genomes of Ascaridida nematodes published previously (Hu et al. 2002, Kim et al. 2006, Li et al. 2008, Xie et al. 2011, Zhao et al. 2018). The base composition, codon usage and nucleotide substitution were analyzed with Mega 7.0. Nucleotide composition skewness was calculated as AT skew = (A − T)/(A + T) and GC skew = (G − C)/(G + C), respectively. The complete mitochondrial genome sequence of *B. sinensis* was deposited in the GenBank DNA database under the accession number PX520829.

### Phylogenetic analysis

To further investigate the phylogenetic relationships of *B. sinensis*, phylogenetic analyses were conducted using maximum-likelihood (ML) tree based on 12 protein-coding genes (PCGs) of 24 species of Ascaridida nematodes with *Wuchereria bancrofti* (NC016186) as the outgroup. The genome sequences from each species were aligned by ClustalW based on PCGs nucleotide sequence and manually trimmed for end-uniform sequences (Tamura et al. 2021). Model Finder (Kalyaanamoorthy et al. 2017) was employed to identify the most suitable evolutionary model (GTR+F + R4). Subsequently, the maximum likelihood (ML) phylogenetic tree using MEGA 7.0 with 1000 bootstrap replicates was constructed (Tamura et al. 2021). The mitochondrial genome map of *B. sinensis* was constructed using the OGDraw online tool (Hao et al. 2024) (https://chlorobox.mpimp-golm.mpg.de/OGDraw.html).

## Results

**Figure 2.**
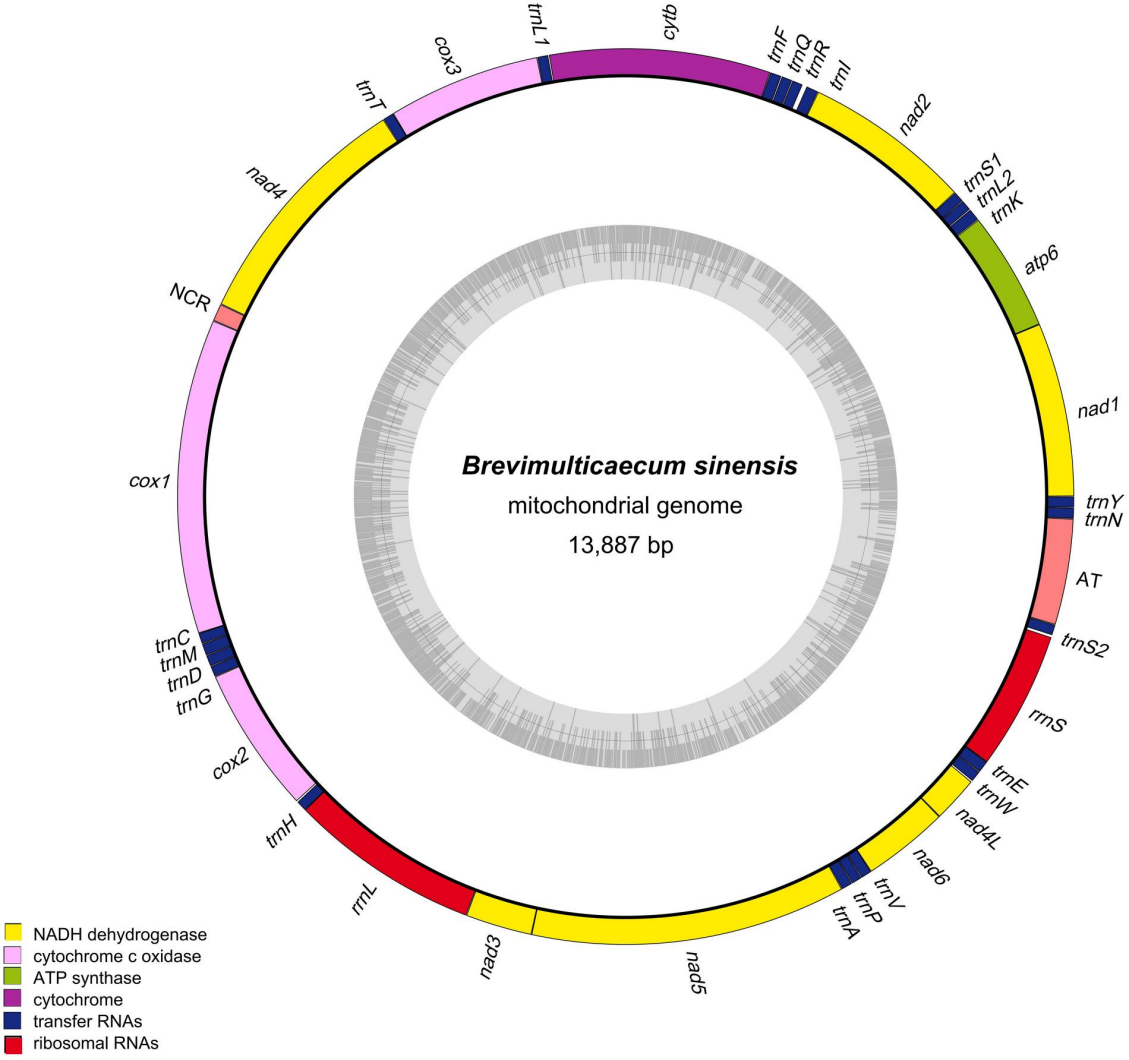
Graphic representation of the mitochondrial genome of *Brevimulticaecum sinensis*. The total length was 13 887 bp in size, contains 12 PCGs (*cox*1-3, *nad*1-6, *nad*4L, *atp*6, *cytb*), 2 ribosomal RNAs (*rrn*S and *rrn*L), 22 transfer RNAs (tRNA), a non coding region (NCR), an AT-rich region. Genes shown in the outer circle are transcribed clockwise, and genes shown in the inner circle are transcribed counterclockwise.

The complete mitogenome of *B. sinensis* was 13 887 bp in size (PX520829), contains 12 PCGs (*cox*1-3, *nad*1-6, *nad*4L, *atp*6, *cytb*), 2 ribosomal RNAs (*rrn*S and *rrn*L), 22 transfer RNAs (tRNA), a non coding region (NCR), an AT-rich region, but lack an *atp*8 gene (Figure 2, Table S1). These genes of *B. sinensis* are all located on the positive strand and transcribed at the same direction. The *rrn*S was located between *trn*E and *trn*S2 (UCN), and *rrn*L was located between *trn*H and *nad*3. AT-rich region was located between genes *trnS* (UCN) and *trnN*, while the NCR was located between genes *nad*4 and *cox*1. The 22 tRNA sequences ranged from 54 to 62 bases in length. Furthermore, nine intergenic spacers totaling 50 bp, and eight overlapping regions totaling 9 bp were scattered throughout the whole genome (Table S2). Consistent with other Ascaridida nematodes, the nucleotide composition for the *B. Sinensis* mitochondrial DNAs was also biased toward A and T (*A* = 26.6%, *T* = 47.8%, *C* = 8.7%, and *G* = 16.9%), and the AT content was 74.4%. AT content of AT-rich region was the highest 89.3% (Table S3). The AT-skew and GC-skew in *B. sinensis* mitogenome were −0.29 and 0.32, respectively.

The overall length of the 12 PCGs in *B. sinensis* mitogenome was 10 325 bp, accounting for 74.35% of the total length of *B. sinensis* mitogenome, encoding 3419 amino acids. Among the 12 PCGs of *B. sinensis*, the longest gene is *nad*5 (1582 bp), and the shortest one is *nad*4L gene (234 bp), same as other Ascaridida nematodes (Xie et al. 2011) (Table S1). All the PCGs utilized typical ATN and TTG codons were observed in *B. sinensis* (Table S1). In detail, among which seven genes (*nad*1, *nad*4, *nad*4L, *nad*5, *cytb*, *cox*2 and *atp*6) started with codon ATT, five genes (*nad*2, *nad*3, *nad*6, *cox*1 and *cox*3) with TTG; Although almost of all PCGs utilized the conventional stop codons TAA (six times) or TAG (four times), the remaining *nad*5 and *nad*2 used incomplete stop codon, such as a single T.

**Figure 3.**
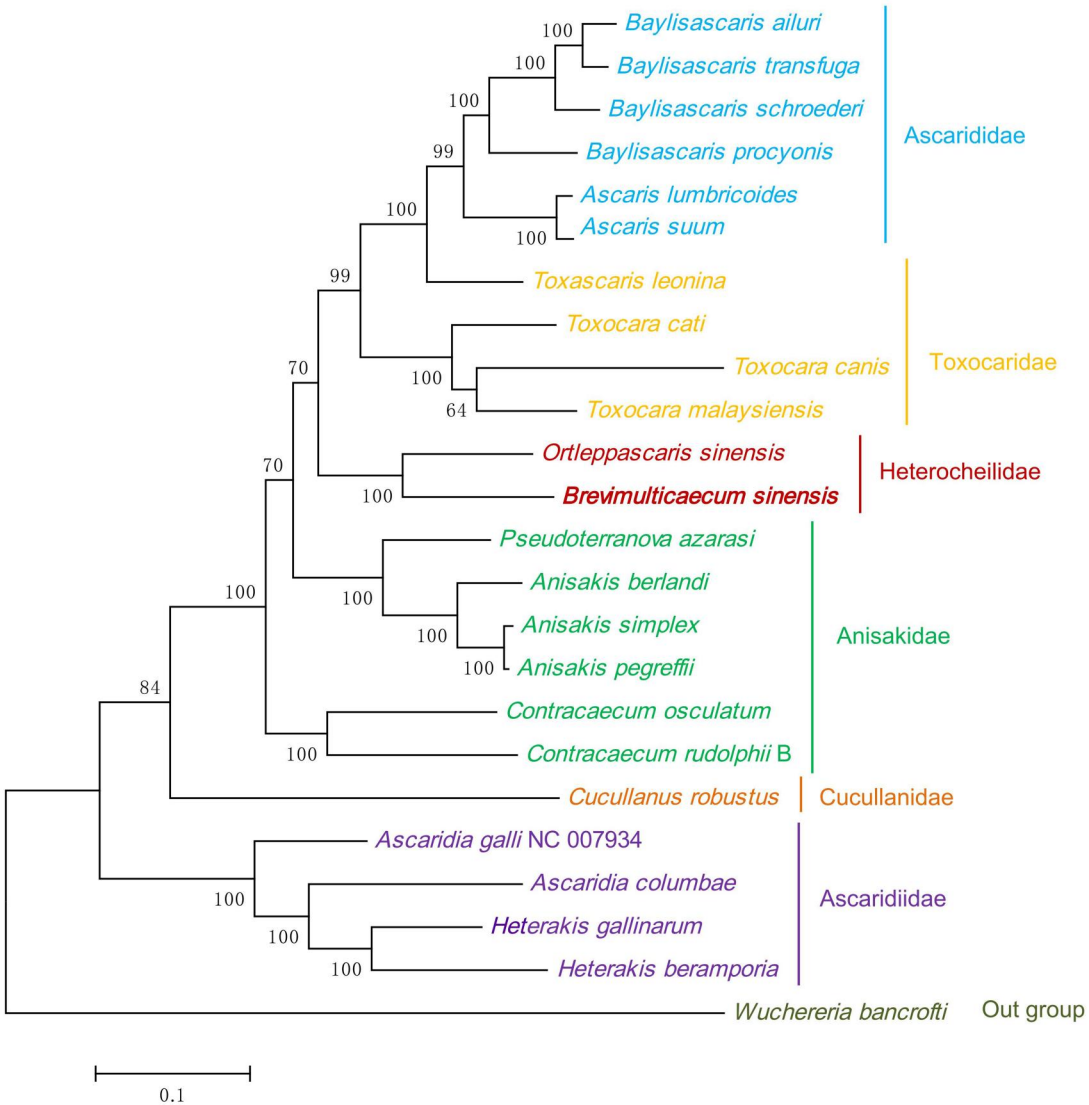
Maximum likelihood (ML) tree based on 12 concatenated mitochondrial genes from 24 species of Ascaridida nematodes. The *Brevimulticaecum sinensis* is marked in red bold font. The following sequences were used: *Baylisascaris ailuri* NC 015925 (Xie et al. 2011), *Baylisascaris transfuga* NC 015924 (Xie et al. 2011), *Baylisascaris schroederi* NC 015927 (Xie et al. 2011), *Baylisascaris procyonis* NC 016200 (Xie et al. 2011), *Ascaris lumbricoides* NC 016198 (Park et al. 2011), *Ascaris suum* NC 001327 (Wolstenholme et al. 1994), *Toxascaris leonina* NC 023504 (Liu et al. 2013), *Toxocara cati* NC 010773 (Li et al. 2008), *Toxocara canis* NC 010690 (Li et al. 2008), *Toxocara malaysiensis* NC 010527 (Li et al. 2008), *Ortleppascaris sinensis* NC 036669 (Zhao et al. 2018), *Pseudoterranova azarasi* NC 027163 (Liu et al. 2015), *Anisakis berlandi* NC 026023 (Kijewska et al. 2015), *Anisakis simplex* NC 007934 (Kim et al. 2005), *Anisakis pegreffii* NC 034329 (Yamada et al. 2017), *Contracaecum osculatum* NC 024037 (Mohandas et al. 2013), *Contracaecum rudolphii* B NC 014870 (Lin et al. 2011), *Cucullanus robustus* NC 016128 (Park et al. 2011), *Ascaridia galli* NC 007934 (Kim et al. 2005), *Ascaridia columbae* NC 021643 (Liu et al. 2013), *Heterakis gallinarum* NC 029839 (Wang et al. 2016), *Heterakis beramporia* NC 029838 (Wang et al. 2016), *Wuchereria bancrofti* NC 016186 (McNulty et al. 2012).

Phylogenetic tree of order Ascaridida was constructed based on the 12 protein-coding genes in the mitochondrial genome sequences using Maximum Likelihood (ML) method. As shown in the phylogenetic tree (Figure 3), there are six families Ascarididae, Toxocaridae, Heterocheilidae, Anisakidae, Cucullanidae, and Ascaridiidae belonged to the order Ascaridida. *B. sinensis* was highly homologous to *Ortleppascaris sinensis* (NC03669) with a 100% bootsrap, forming a monophyletic group belonging to the family Heterocheilidae. Furthermore, the nematode species examined in the ML tree of this study had high bootstrap values for inclusion in the order Ascaridida, thereby validating their taxonomic cohesion.

## Discussion and conclusion

The mitochondrial genome of *B. sinensis* is 13 887 bp in length, comprises 36 genes, but lack an *atp*8 gene. The mitochondrial arrangement of *B. sinensis* was similar as those of the Ascaridae, Toxocaridae, Anisakidae, Heterocheilidae, and Strongylidae, but different from those of Cucullanidae and Ascaridiidae (Hu et al. 2002, Kim et al. 2006, Li et al. 2008, Zhao et al. 2018, Hao et al. 2024). The mitochondrial arrangement of *B. sinensis* was in full accord with the mode of GA7 (gene arrangement, GA) described by Yatawara et al. (2010). Especially the arrangement of 12 PCGs, 2 ribosomal RNAs, 22 tRNAs, one NCR and an AT-rich region are completely consistent to the *O. sinensis*, there are belong to the same family Heterocheilidae. Differences and commonalities in the mitogenomes of different nematodes can be used to analyze the phylogenetic evolution and population genetics of nematode species (Shuai et al. 2023). The lack of the *atp*8 gene does not represent a functional defect but rather reflects genomic streamlining, which may be compensated by the structure of other subunits to maintain ATP synthase function, demonstrating an efficient evolutionary strategy (Zeng et al. 2024).

The nucleotide composition of *B. sinensis* mitochondrial DNA was also biased toward A and T, which is consistent with that of other Ascaridida nematodes. In detail, the T base content was the highest and the C base content was the lowest value in each gene sequence. In general, Ascaridida nematodes have an AT specific bias and rejection of C in nucleotide composition (Xie et al. 2011). Although the exact mechanism causing the AT bias in mtDNA remains unknown, such situation could be the result of inversions of the AT-rich regions and replication origin (Zhao et al. 2018). This AT specific bias may enable nematodes to enhance environmental adaptability by reducing replication energy consumption and improving transcription efficiency (Zeng et al. 2024). As a result of base composition of the nematodes mtDNA has obvious A and T bias, the genetic codes of nematodes mtDNA is given priority to codons rich in A and T (Gao et al. 2017). All the PCGs utilized typical ATN and TTG codons were observed in *B. sinensis.* This AT-enriched codon usage preference can optimize translation efficiency, enabling more efficient expression of mitochondrial proteins under specific physiological conditions (Li et al. 2023).

Moreover, the AT-rich region of the *B. sinensis* mtDNA contained three large tracks of (AT)_10_, (AT)_12_ and (AT)_23_ dinucleotide within a total of 90 bp. Similar multiple TA repeats have been described in the AT-rich region of the mitochondrial genomes for *A. simplex* and hookworm nematodes *Ancylostoma duodenale* and *Necator americanus* (Hu et al. 2002, Kim et al. 2006). Meanwhile, the AT-rich region is the most variable segment of the genome in both length and nucleotide sequence among the mitochondrial genomes of Ascaridida nematodes. As a result, the AT-rich region has been recognized to serve as essential elements involved in the initiation of replication and transcription of mitogenome (Hao et al. 2024).

Through phylogenetic analysis, we found a close relationship between *B. sinensis* and *O. sinensis*, both of which belong to the family Heterocheilidae. Zhao et al. (2018, 2026) also concluded that they belongs to the family Heterocheilidae upon the morphologic characteristics using light and scanning electron microscopic examination and molecular marker of internal transcribed spacers (ITS) of *B. sinensis* and *O. sinensis*. It is necessary to sequence more mtNDAs of Heterocheilidae nematodes in the future to further elucidate the value of mitochondrial genomes in species evolutionary research. This study provides the first comprehensive analysis of the complete mitochondrial genome of *B. sinensis*, filling a critical gap in mitochondrial genomic data for this species. The mitochondrial genome of *B. Sinensis* provides more accurate DNA molecular markers, enabling effective differentiation nematode populations with similar morphology but significant genetic divergence, thereby enhancing the accuracy of species identification and system taxonomy.

## Supplementary Material

Supplemental Material

## Data Availability

The genome sequence data that support the findings of this study are openly available in GenBank of NCBI at under accession number PX520829.
