# Supplementary material for: The complete mitochondrial genome of Brevimulticaecum sinensis (Nematoda: Heterocheilidae), the first representative of the genus Brevimulticaecum
Source: Mitochondrial DNA B Resour. 2026 Jun 19;11(7):869–73. doi: 10.1080/23802359.2026.2688608 (PMC13288723; doi:10.1080/23802359.2026.2688608)
Supplement: Supplemental Material [file TMDN_A_2688608_SM4842.docx]

Table S1 PCR primer of *Brevimulticaecum sinensis* for amplication of mitochondrial genome

| Region | Name | Sequence（5`to 3`） | Reference |
| --- | --- | --- | --- |
| cox1 | LCO1490 | GGTCAACAAATCATAAAGATATTGG | Folmer et al., 1994 |
|  | HCO2198 | TAAACTTCAGGGTGACCAAAAAATCA | Folmer et al., 1994 |
| rrnS | rrnS-F1 | GTTCCAGAATAATCGGCTAGAC | Zhao et al., 2017 |
|  | rrnS-R1 | GGCAATTGATGGATGATTTGTACC | Zhao et al., 2017 |
| nad1 | nad1F | TGAGCGTCATTTNYTNGG | Zhao et al., 2017 |
|  | nad1R | AANGGNGCHCGRTTYARYTC | Zhao et al., 2017 |
| cox3 | COX3F | GGTDAATCCTTTTGGNGTNCC | Zhao et al., 2017 |
|  | COX3 R | GGCAAAYTCYAAMCCNARRTG | Zhao et al., 2017 |
| nad3-nad5 | Nad3-F | TGATTTGGAGGTGGTCATA | Zhao et al., 2017 |
|  | Nad5-R | CATAGARAACCCYATCTGNGA | Zhao et al., 2017 |
| nad1-nad2 | Nad1-F | AGTTGGGGCTATTCGTGCTA | Zhao et al., 2017 |
|  | Ile-H | CTCCGCCTTATCAAGACGACAT | Zhao et al., 2017 |
| nad2-cytb | nad2-cytb-F | GCGTGGCTCCTTTTCATT | Zhao et al., 2017 |
|  | nad2-cytb-R | CACTCWGGNACAATATGNACAGG | Zhao et al., 2017 |
| cytb-cox3 | cytb-cox3-F | ACGGTCTGGTTGTTACTTTGCC | This study |
|  | cytb-cox3-R | GTGACTGATACACCCCTTCTTA | This study |
| cox3-cox1 | cox3-cox1-F | GGGTTCCTTTGCTAAACACTA | This study |
|  | cox3-cox1-R | GCCAAATCTACTCTACTTCCC | This study |
| cox1-nad3 | cox1-nad3-F | CAGTATTGGCTGGAGCGATT | This study |
|  | cox1-nad3-R | TGGACCAATGAACTCACAGG | This study |
| nad5-rrnS | nad5-rrnS-F | TGTGAGTTCATTGGTCCATAGT | This study |
|  | nad5-rrnS-R | CAGGTTCCCCTACCCCTACTT | This study |
| rrnS-nad1 | rrnS-nad1-F | ATCTTTGGAGGTTGAGTAG | This study |
|  | rrnS-nad1-R | CCTTACAACACCCCTTACT | This study |

Table S2 Organization of the *Brevimulticaecum sinensis* mitochondrial genome

| Gene | Position | Size | Spacer(+)  Overlap(-) | Start codon | Stop codon | AA | anticodon | AT-skew | GC-skew |
| --- | --- | --- | --- | --- | --- | --- | --- | --- | --- |
| *nad1* | 1-873 | 873 | 1(-) | ATA | TAA | 289 |  | -0.38 | 0.37 |
| *atp6* | 873-1472 | 600 | 1(+) | ATA | TAG | 198 |  | -0.30 | 0.37 |
| *trnK* | 1474-1535 | 62 | 2(+) |  |  |  | TTT |  |  |
| *trnL2*UUR | 1538-1592 | 55 | 0 |  |  |  | TAA |  |  |
| *trnS1*AGN | 1593-1647 | 55 | 0 |  |  |  | TCT |  |  |
| *nad2* | 1648-2491 | 844 | 0 | TTG | T | 280 |  | -0.35 | 0.42 |
| *trnI* | 2492-2553 | 62 | 21(+) |  |  |  | GAT |  |  |
| *trnR* | 2575-2629 | 55 | 1(+) |  |  |  | ACG |  |  |
| *trnQ* | 2631-2685 | 55 | 2(+) |  |  |  | TTG |  |  |
| *trnF* | 2688-2745 | 58 | 0 |  |  |  | GAA |  |  |
| *cytb* | 2746-3852 | 1107 | 3(+) | ATA | TAA | 367 |  | -0.36 | 0.32 |
| *trnL1*CUN | 3856-3910 | 55 | 0 |  |  |  | TAG |  |  |
| *cox3* | 3911-4678 | 768 | 1(-) | TTG | TAG | 254 |  | -0.43 | 0.33 |
| *trnT* | 4678-4732 | 55 | 0 |  |  |  | TGT |  |  |
| *nad4* | 4733-5962 | 1230 | 0 | ATG | TAA | 408 |  | -0.35 | 0.16 |
| NCR | 5963-6051 | 89 | 0 |  |  |  |  |  |  |
| *cox1* | 6052-7629 | 1578 | 1(-) | TTG | TAG | 524 |  | -0.31 | 0.30 |
| *trnC* | 7629-7682 | 54 | 0 |  |  |  | GCA |  |  |
| *trnM* | 7683-7743 | 61 | 0 |  |  |  | CAT |  |  |
| *trnD* | 7744-7801 | 58 | 0 |  |  |  | GTC |  |  |
| *trnG* | 7802-7857 | 56 | 0 |  |  |  | TCC |  |  |
| *cox2* | 7858-8595 | 738 | 5(+) | ATA | TAA | 244 |  | -0.27 | 0.32 |
| *trnH* | 8601-8655 | 55 | 1(-) |  |  |  | GTG |  |  |
| *rrnL* | 8655-9611 | 957 | 0 |  |  |  |  |  |  |
| *nad3* | 9612-9947 | 336 | 1(-) | TTG | TAA | 110 |  | -0.39 | 0.69 |
| *nad5* | 9947-11528 | 1582 | 0 | ATT | T | 526 |  | -0.35 | 0.31 |
| *trnA* | 11529-11585 | 57 | 0 |  |  |  | TGC |  |  |
| *trnP* | 11586-11639 | 54 | 0 |  |  |  | TGG |  |  |
| *trnV* | 11640-11695 | 56 | 1(-) |  |  |  | TAC |  |  |
| *nad6* | 11695-12129 | 435 | 1(-) | TTG | TAA | 143 |  | -0.43 | 0.30 |
| *nad4L* | 12129-12362 | 234 | 5(+) | ATA | TAG | 76 |  | -0.30 | 0.49 |
| *trnW* | 12368-12424 | 57 | 0 |  |  |  | TCA |  |  |
| *trnE* | 12425-12486 | 62 | 1(-) |  |  |  | TTC |  |  |
| *rrnS* | 12486-13181 | 696 | 10(+) |  |  |  |  |  |  |
| *trnS2*UCN | 13192-13245 | 54 | 0 |  |  |  | TGA |  |  |
| AT | 13246-13777 | 532 | 0 |  |  |  |  |  |  |
| *trnN* | 13778-13832 | 55 | 0 |  |  |  | GTT |  |  |
| *trnY* | 13833-13887 | 55 | 0 |  |  |  | GTA |  |  |

AT: AT rich region. NCR: Non-coding region.

Table S3 Length and AT contents (%) of mitochondrial genome of *Brevimulticaecum sinensis*

|  | length(bp) | A | C | T | G | A+T |
| --- | --- | --- | --- | --- | --- | --- |
| mtDNA | 13887 | 26.6 | 8.7 | 47.8 | 16.9 | 74.4 |
| *nad*1 | 873 | 22.8 | 8.4 | 50.5 | 18.3 | 73.3 |
| *nad*2 | 844 | 25.7 | 6.2 | 53.0 | 15.2 | 78.7 |
| *nad*3 | 336 | 24.1 | 3.3 | 54.5 | 18.2 | 78.6 |
| *nad*4 | 1230 | 23.9 | 11.1 | 49.8 | 15.3 | 73.7 |
| *nad*4L | 234 | 27.8 | 5.1 | 52.1 | 15.0 | 79.9 |
| *nad*5 | 1582 | 24.9 | 8.2 | 51.4 | 15.5 | 76.3 |
| *nad*6 | 435 | 22.1 | 8.0 | 54.9 | 14.9 | 77.0 |
| *cox*1 | 1578 | 23.4 | 11.1 | 44.8 | 20.7 | 68.3 |
| *cox*2 | 738 | 24.8 | 10.8 | 43.5 | 20.9 | 68.3 |
| *cox*3 | 768 | 20.1 | 9.8 | 50.8 | 19.4 | 70.8 |
| *cytb* | 1107 | 22.8 | 9.8 | 48.5 | 19.0 | 71.3 |
| *atp*6 | 600 | 25.8 | 8.2 | 48.2 | 17.8 | 74.0 |
| *rrn*L | 957 | 33.4 | 6.6 | 45.9 | 14.1 | 79.3 |
| *rrn*S | 696 | 34.3 | 10.2 | 37.4 | 18.1 | 71.7 |
| tRNAs | 1246 | 33.3 | 8.9 | 41.3 | 16.5 | 74.6 |
| AT-rich region | 532 | 40.8 | 4.9 | 48.5 | 5.8 | 89.3 |
| NCR | 89 | 30.3 | 5.6 | 49.4 | 14.6 | 79.8 |
